# Safety climate perception for staff nurses in an ICU

**DOI:** 10.1186/cc12439

**Published:** 2013-03-19

**Authors:** RL Rivera-Romero, M Torres-Campos, MJ Delgado-Amaya, E Curiel-Balsera, JG Quesada-García

**Affiliations:** 1 Hospital Regional Carlos Haya, Málaga, Spain

## Introduction

When we talk about safety culture, we speak of being aware that things can go wrong. We must be able to recognize mistakes and learn from them, sharing that information fairly and impartially to try to prevent its recurrence. Organizations such as the Agency for Healthcare Research and Quality (AHRQ) have developed tools to help organizations measure their safety culture and there is little information about our country.

## Methods

A descriptive survey study. We sent the Spanish version of the questionnaire on patient safety culture (AHRQ) to the nursing staff of a polyvalent ICU of 42 beds in a tertiary hospital.

## Results

The questionnaire was sent to 179 nurses, receiving correctly answered 88 surveys (response rate of 49.16%). On a scale of 0 to 10, 6.97 points was obtained to estimate the safety climate for staff respondents. The item best scored was teamwork in the unit (65.9%). Detected as a fortress, 'communication between nurses at shift changes' (76.1% positive responses). The worst rating was obtained in the section on human resources, followed by management support in the field of patient safety.

## Conclusion

The perception of safety culture in an ICU by nursing staff is far from optimal levels. The team work dimension was identified as the most valued by workers, with the transmission of information on shift changes the most valued item.

